# A Neurological Outpatient Clinic for Patients With Post-COVID-19 Syndrome — A Report on the Clinical Presentations of the First 100 Patients

**DOI:** 10.3389/fneur.2021.738405

**Published:** 2021-09-16

**Authors:** Fabian Boesl, Heinrich Audebert, Matthias Endres, Harald Prüss, Christiana Franke

**Affiliations:** ^1^Department of Neurology, Charité – Universitätsmedizin Berlin, corporate member of Freie Universität Berlin and Humboldt-Universität zu Berlin, Berlin, Germany; ^2^Center for Stroke Research Berlin, Berlin, Germany; ^3^German Center for Neurodegenerative Diseases (DZNE), Berlin, Germany; ^4^Excellence Cluster NeuroCure, Berlin, Germany; ^5^German Centre for Cardiovascular Research (DZHK), Berlin, Germany

**Keywords:** post-COVID-19, long-COVID-19, neurology, outpatient, SARS-CoV-2

## Abstract

**Background and Objectives:** Neurological and psychiatric symptoms are frequent in patients with post-COVID-19 syndrome (PCS). Here, we report on the clinical presentation of the first 100 patients who presented to our PCS Neurology outpatient clinic ≥12 weeks after the acute infection with SARS-CoV-2. To date, PCS is only defined by temporal connection to SARS-CoV-2 infection. Identification of clinical phenotypes and subgroups of PCS is urgently needed.

**Design:** We assessed clinical data of our first 100 ambulatory patients regarding clinical presentations; self-questionnaires focusing on daytime sleepiness, mood, and fatigue; and a screening assessment for detecting cognitive impairment.

**Results:** A total of 89% of the patients presenting to the Neurology outpatient clinic had an initially mild course of COVID-19 and had not been hospitalized. The majority of the patients were female (67 vs. 33% male). The most frequent symptom reported was cognitive impairment (72%). There were 30% of patients who reported cognitive deficits and scored below 26 points on the Montreal Cognitive Assessment Scale. Fatigue (67%), headache (36%), and persisting hyposmia (36%) were also frequently reported; 5.5% of all patients showed signs of severe depression.

**Discussion:** To our knowledge, this is the first report of patient data of a PCS Neurology outpatient clinic. Neurological sequelae also exist for more than 3 months after mainly mild SARS-CoV-2 acute infections. The reported symptoms are in accordance with recently published data of hospitalized patients.

## Introduction

Signs and symptoms that develop during or after an infection with SARS-CoV-2 and continue for more than 12 weeks and are not explained by an alternative diagnosis are defined as post-COVID-19 syndrome (PCS) (1). Recent publications report a number of neurological and psychiatric symptoms including fatigue, cognitive impairment, insomnia, myalgia, headache, vertigo, anxiety, and depression (2–5). The most frequently reported PCS symptoms, analyzed in a Swedish cohort study in health care professionals with mild SARS-CoV-2 acute infection, were of neurological nature and led to continuous functional impairment in work, social, and home life (6). A retrospective cohort study showed that neurological and psychiatric disorders were more common after COVID-19 compared to other respiratory tract infections and influenza (7). Here, we present clinical data of ambulatory patients from a specialized PCS outpatient clinic for patients with neurological manifestations.

## Methods

In September 2020, we established a PCS outpatient clinic focusing on neurological symptoms in the aftermath of COVID-19. Patients presenting to our outpatient clinic did not suffer from neurological diseases prior to the infection with SARS-CoV-2 and were referred from general practitioners or neurologists working in the outpatient setting. Between September 2020 and April 2021, patients admitted to our outpatient clinic were interviewed and examined by a physician who specialized in Neurology. All patients had a confirmed diagnosis of SARS-Cov-2 infection (either positive PCR testing for SARS-CoV-2-RNA during the acute infection or positive testing for SARS-CoV-2 antibodies). Self-questionnaires for further assessment of daytime sleepiness (Epworth Sleepiness Scale, ESS), mood (Beck Depression Inventory Version I, BDI), and fatigue (Fatigue Severity Scale, FSS) were applied. The Montreal Cognitive Assessment Scale (MoCA) was used to detect cognitive impairment. Tables 1, 2 show an overview of patient characteristics, clinical symptoms, and results of self-questionnaires and cognitive testing of our cohort.

**Table 1:** Characteristics of patients presenting to the neurological Post-COVID-19 outpatient clinic.

	**Total**	**Female**	**Male**
Number of patients	100	67	33
Mean age (years)	45.8	45.2	46.9
- Range	20–79	21–68	20–79
Mean time between positive SARS-CoV-2 testing and presentation in outpatient clinic (days)	184.5	186.9	179.7
- Range (days)	85–426	85–426	91–369
Mild COVID-19 course	89	61 (91.0%)	28 (84.8%)
Severe COVID-19 course	11	6 (9.0%)	5 (15.2%)
**Symptoms**			
Cognitive impairment	72	45 (67.2%)	27 (81.8%)
Fatigue	67	49 (73.1%)	18 (54.5%)
Headache	36	24 (35.8%)	12 (36.4%)
Myalgia	21	15 (22.4%)	6 (18.2%)
Limb pain	9	5 (7.5%)	4 (12.1%)
Other pain	17	12 (17.9%)	5 (15.2%)
Hyposmia	36	27 (40.3%)	9 (27.3%)
Vertigo	20	15 (22.4%)	5 (15.2%)

**Table 2:** Evaluation of self-questionnaires and results of MoCA of patients presenting to the neurological post-COVID-19 outpatient clinic.

	**Total**	**Female**	**Male**
ESS (mean; 0–24 points; *n* = 92)	8.6	8.3	9.2
- Normal daytime sleepiness (0–10 points)	61 (66.3%)	43 (67.2%)	18 (64.3%)
- Mild to moderate excessive daytime sleepiness (11–15 points)	25 (27.2%)	17 (26.5%)	8 (28.6%)
- Severe excessive daytime sleepiness (16–24 points)	6 (6.5%)	4 (6.3%)	2 (7.1%)
BDI (mean; 0–63 points; *n* = 91)	13.5	14.1	12.3
- No depression (0–9 points)	35 (38.5%)	22 (34.9%)	13 (46.4%)
- Mild depression (10–19 points)	34 (37.4%)	25 (39.7%)	9 (32.1%)
- Moderate depression (20–29 points)	17 (18.7%)	11 (17.5%)	6 (21.4%)
- Severe depression (30–63)	5 (5.5%)	5 (7.9%)	0
FSS (mean; 1–7 points; *n* = 91)	5.0	5.1	4.7
- No impairment due to fatigue (1–3 points)	18 (19.8%)	13 (20.3%)	5 (18.5%)
- Impairment due to fatigue (4–7 points)	73 (80.2%)	51 (79.7%)	22 (81.5%)
MoCA (mean; 0–30 points; *n* = 72)	26.7	26.9	26.3
- Normal (26–30 points)	50 (69.4%)	32 (71.1%)	18 (66.7%)
- Pathologic (≤ 25 points)	22 (30.6%)	13 (28.9%)	9 (33.3%)

## Results

The mean age was 45.8 years (range: 20–79 years). Time between positive testing for SARS-CoV-2 and first presentation in our outpatient clinic varied from 85 to 426 days (mean, 184.5 days). Considerably more female (67%) than male (33%) patients were referred. The majority of the patients (89%) reported a mild course of acute SARS-CoV-2 infection with no need of oxygen supplementation or admission to a hospital. The most frequent symptoms were cognitive impairment (72%), fatigue (67%), headache (36%), and persisting hyposmia (36%). Further symptoms were myalgia (21%), vertigo (20%), and other pain syndromes (17%) including limb pain (9%). A total of 30% of patients with cognitive deficits showed pathologic MoCA scores (≤ 25/30 points). In 6.5%, the ESS signified a severe excessive daytime sleepiness. Female patients tended to have a higher BDI score than male patients (14.1 vs. 12.3 points); 5.5% of the patients had severe depression, and 38.5% showed scores compatible with no depression. There were 80.2% of patients who reported significant impairment of their daily life due to fatigue measured by the FSS.

## Discussion

We report data of patients who presented to our PCS Neurology outpatient clinic due to persisting neurological symptoms ≥12 weeks after the acute infection with SARS-CoV-2. To our knowledge, this is the first report of patient data of a PCS Neurology outpatient clinic. The percentage of patients who had an initial mild clinical course of the disease was slightly higher in comparison to the overall reported percentage of 81% mild disease courses in COVID-19 (8). Interestingly and according to recently published data of hospitalized patients, residual neurological complaints expressed by our cohort predominantly involved cognitive impairment, fatigue, and headache (2–5, 9). Thirty percent of patients reporting cognitive deficits showed pathologic MoCA scores resulting in further diagnostics including imaging, cerebrospinal fluid diagnostic, and neuropsychological assessment (10, 11). These additional data need evaluation in a sufficient number of patients with PCS to draw further conclusions.

Pathophysiological mechanisms of the described neurological deficits remain largely unknown for PCS. To date, biomarkers and imaging findings are not identified. For severe COVID-19 courses, inflammation, hypoxemia, and vascular mechanisms might contribute to etiopathogenesis. Additionally, an autoantibody production triggered by SARS-CoV-2 is postulated (12, 13). While male sex is proven to be a risk factor for severe COVID-19 acute disease course, women seem to be more vulnerable to develop PCS. This finding is in line with previously published data (14). This might be due to reporting bias, since women tend to seek medical advice more commonly. Another possible explanation is that women are more frequently prone to autoimmune disorders (15).

Anxiety and depression have been reported in patients with PCS; however, severe depression was only presented in 5% in our cohort. Overall prevalence of depressive symptoms in the German population is reported to be 8.1% compared to 61.5% in our cohort (16). The etiology of these neuropsychiatric symptoms needs further evaluation, but might be partially attributed to reactive mood alterations suitable for the prolonged impairment. As previously reported, fatigue in PCS has a high impact on the daily life of patients (14). Prevalence of fatigue in our patients was over four times higher than in a reported cohort of healthy individuals (18%) and higher than in a cohort of patients with multiple sclerosis (69 vs. 80.2%) (17). Distinguishing fatigue from excessive daytime sleepiness, the latter was only present in one third of our patients. This is a markedly higher prevalence than that reported of the general populations of several countries (18–21). Pathophysiological mechanisms of fatigue and sleep disorders including daytime sleepiness are under current investigation. Mannose-binding lectin deficiency and elevated interleukin-8 (IL-8) levels have been reported in patients suffering from fatigue and might serve as potential biomarkers (22).

We see a high demand of neurological PCS outpatient care, and rising numbers of PCS patients are to be expected. For the best medical care of these patients, thoroughly assessed data of patients in the outpatient setting are needed. Close interdisciplinary collaboration between pneumologists, cardiologists, psychiatrist, psychosomatic physicians, and neurologists should be established for the most beneficial diagnostic workup and the development of management plans for PCS patients, which are currently unavailable. In the absence of medical therapeutic options, the valuable expertise of physiotherapists, occupational therapist, psychotherapists, and neuropsychologists is crucial in the treatment of PCS. Availability of specialized PCS rehabilitation centers and interdisciplinary treatment options is strongly needed. A broad organized network of structured patient education might prevent misinformation of affected patients in need. Further evaluation and in-depth studies of the pathophysiological mechanisms of PCS conditions are urgently needed to offer specific therapeutic regimens in the future.

## Limitations

This report is providing data of a single neurological outpatient clinic that was established to offer specialized medical care to patients with neurological manifestations of PCS. Therefore, comparison to a cohort of PCS patients without neurological manifestations was not possible. Comparison of our data with data of other neurological PCS outpatient clinics is needed to validate our data and allow generalization.

## Data Availability Statement

The original contributions presented in the study are included in the article/supplementary material, further inquiries can be directed to the corresponding author/s.

## Ethics Statement

The studies involving human participants were reviewed and approved by Charité's Ethics Committee Chairperson: Ms. Dr. Orzechowski Number: EA2/066/20 Charité–Universtity Medicine Berlin. The patients/participants provided their written informed consent to participate in this study.

## Author Contributions

FB and CF conceptualized the project, had a major role in data collection, and wrote the manuscript. HA, ME, and HP conceptualized the project. All authors critically revised the manuscript for important intellectual content and approved the final draft.

## Funding

We acknowledge support from the German Research Foundation (DFG, FR 4479/1-1) and the Open Access Publication Fund of Charité – Universitätsmedizin Berlin.

## Conflict of Interest

The authors declare that the research was conducted in the absence of any commercial or financial relationships that could be construed as a potential conflict of interest.

## Publisher's Note

All claims expressed in this article are solely those of the authors and do not necessarily represent those of their affiliated organizations, or those of the publisher, the editors and the reviewers. Any product that may be evaluated in this article, or claim that may be made by its manufacturer, is not guaranteed or endorsed by the publisher.
